# Association Between Hematological Parameters and Iron Metabolism Response After Marathon Race and *ACTN3* Genotype

**DOI:** 10.3389/fphys.2019.00697

**Published:** 2019-06-11

**Authors:** Ana Paula Renno Sierra, Rodrigo Assunção Oliveira, Elton Dias Silva, Giscard Humberto Oliveira Lima, Marino Pereira Benetti, Maria Augusta Pedanti Kiss, Carlos Anibal Sierra, Nabil Ghorayeb, Jane T. Seto, João Bosco Pesquero, Maria Fernanda Cury-Boaventura

**Affiliations:** ^1^ Department of Biodynamics of Human Movements, School of Physical Education and Sports, University of São Paulo, São Paulo, Brazil; ^2^ Sports Cardiology Department, Dante Pazzanese Institute of Cardiology, São Paulo, Brazil; ^3^ Department of Interdisciplinary in Health Sciences, Institute of Physical Activity and Sports Sciences, Cruzeiro do Sul University, São Paulo, Brazil; ^4^ Department of Biophysics, Federal University of São Paulo, São Paulo, Brazil; ^5^ Department of Movement, Human and Health Sciences, Program of Human Movement and Sport Sciences, University of Rome “Foro Italico”, Rome, Italy; ^6^ Neuromuscular Research, Murdoch Children’s Research Institute, Melbourne, VIC, Australia

**Keywords:** long-distance exercise, iron metabolism, polymorphism, hematological parameters, actinin-3

## Abstract

α-Actinin-3 (*ACTN3* R577X, rs.1815739) polymorphism is a genetic variation that shows the most consistent influence on metabolic pathway and muscle phenotype. XX genotype is associated with higher metabolic efficiency of skeletal muscle; however, the role of *ACTN3* polymorphism in oxygen transport and utilization system has not yet been investigated. Therefore, the aim of this study was to determine the influence of *ACTN3* polymorphisms on hematological and iron metabolism response induced by marathon race. Eighty-one Brazilian amateur male endurance runners participated in the study. Blood samples and urine were collected before; immediately after; and 1, 3, and 15 days after the marathon race. Urine, hematological parameters, iron metabolism, and *ACTN3* genotyping analyses were performed. The marathon race induced a decrease in erythrocytes, Hb, and Ht, and an increase in hematuria, creatinine, myoglobin, red cell distribution width, mean corpuscular hemoglobin concentration, mean corpuscular hemoglobin, direct and indirect bilirubin and erythropoietin. Moreover, an elevation immediately or 1 day after the marathon race follows a reduction 3 or 15 days after the marathon race were observed on transferrin saturation and iron and transferrin levels. Hematological parameters and iron metabolism changes induced by marathon race were not observed in XX genotypes. Hematuria and decreased erythrocytes, Hb, Ht, and iron and transferrin levels were observed only in RR and/or RX genotypes but not in XX genotypes. The percentage of runners with hematuria, leukocyturia, iron deficiency, creatinine, myoglobin, and bilirubin imbalance was higher in RR compared to XX genotypes. *ACTN3* polymorphism is associated with iron metabolism and hematological responses after endurance exercise. Despite these results being based on a small sample, they highlight a protective role of the XX genotype on hematological and renal changes induced by long-distance exercise. Therefore, these findings should be further replicated.

## Introduction

Iron deficiency is associated with impairment in the transport and use of oxygen, and consequently increases the risk to sports anemia and may affect athletic performance (Peeling et al., 2008, 2014). The potential mechanisms involved in iron deficiency induced by long-distance exercise are intravascular and extravascular hemolysis, hematuria, and sweat and gastrointestinal iron loss and inflammation (Kobayashi et al., 2014; Peeling et al., 2014). Hemolysis is caused by many factors such as oxidative stress, inflammation, erythrocyte deformability, hemodilution, and mechanical rupture from foot strike or from compression of muscle on capillaries (Telford et al., 2003; Robinson et al., 2006; Yusof et al., 2007; Lippi et al., 2012; Mairbaurl et al., 2013; Peeling et al., 2014; Robach et al., 2014). High body temperature and catecholamine levels, hypotension after ischemia, foot strike, and bladder trauma also contribute to hematuria after intense exercise (Bellinghieri et al., 2008; Shephard, 2016).

Previous studies suggest that hemolysis is a heritable trait and is correlated with many energy metabolites (Van 't Erve et al., 2015). Hypoxia is the major stimulus for the release of ATP from erythrocytes, hemolysis, and hematuria (Sikora et al., 2014). *ACTN3* (α-Actinin-3) R577X polymorphism has consistently been shown to influence metabolic pathway and muscle performance (MacArthur and North, 2004, 2007; Eynon et al., 2013; Lee et al., 2016). The sarcomeric α-actinins (ACTN2 and ACTN3) are major components of the muscle Z-line but expression of ACTN3 is restricted to Z-lines of fast-twitch fibers while ACTN2 is expressed in all fibers (North et al., 1999; Mills et al., 2001; Eynon et al., 2013). *ACTN3* R577X polymorphism is a single-nucleotide polymorphism (SNP) at codon 577 (rs 1,815,739) due to replacement of an arginine (R) with a stop codon (X); homozygosity for the X allele results in a lack of production of functional ACTN3 protein (North et al., 1999; Mills et al., 2001; Eynon et al., 2013). Approximately, 18% of Caucasians present XX genotype and are completely deficient in ACTN3 protein (Eynon et al., 2014). The functional properties of ACTN2 and ACTN3 seem to be determined by capacity to interact with key proteins involved in biological processes (Seto et al., 2011a,b; Lee et al., 2016). ACTN3 deficiency and XX genotype has been associated with decreased muscle strength, muscle mass, and fast-twitch fiber, however, to improve the metabolic efficiency of skeletal muscle in humans and in the *Actn3* knockout mouse model (Seto et al., 2013; Erskine et al., 2014; Norman et al., 2014; Kikuchi and Nakazato, 2015; Houweling et al., 2018; Del Coso et al., 2019). Our hypothesis is that the metabolic efficiency attributed to XX genotype may improve parameters of oxygen transport and utilization system (red blood cells and iron metabolism) after long-distance exercise. Blood flow and oxygen demand increase in muscle cells during exercise, promoting changes in some metabolites such as 2,3-diphosphoglycerate (2,3-DPG) and adenosine triphosphate (ATP), H^+^, CO_2_, Cl^−^, and/or leading to hypoxia that modulates oxygen transport and utilization system and hematuria (renal hypoxia) (Mairbaurl, 2013).

On this basis, the aim of this study is to determine the influence of *ACTN3* R577X polymorphisms on hematological parameters and iron metabolism response induced by amateur marathon running.

## Materials and Methods

### Subjects

Eighty-one Brazilian amateur male endurance runners that completed the São Paulo International Marathon 2015 participated in the current study. The recruitment of the volunteers was performed by the São Paulo International Marathon Organization (2015) by mailing. Runners were randomized after medical history and examination and training history. The noninclusion criteria were: the use of medication for cardiac, metabolic, pulmonary, or kidney injury; use of alcohol or any kind of drugs; pathologies including systemic arterial hypertension, liver, kidney, metabolic, inflammatory, or neoplastic diseases; not having participated in a half-marathon or marathon previously; and training volume less than 40 km per week. Subjects were informed of the experimental procedures and possible risks and signed the written informed consent before participating. The study and written informed consent were approved by the Ethics Committee of Dante Pazzanese Institute of Cardiology, Brazil (Permit Number: 979/2010), in accordance with the Declaration of Helsinki.

São Paulo International Marathon 2015 was performed on 17 May at 08:00 am. Every 2–3 km during the run, water was allowed *ad libitum*; sports drinks on 18 and 36 km; and potato and gel on 30 km. The weather parameters at São Paulo International Marathon in 2015 between 8 am and 2 pm were: average temperature was 19.8°C (maximum = 22.6°C, minimum = 16.7°C) and average relative humidity was 72.8% (maximum = 86%, minimum = 61%) (National Institute of Meteorology, Ministry of Agriculture, Livestock and Supply).

Total body mass (kg), height (cm), and body mass index (BMI, kg/m^2^) were measured according to the International Society for the Advancement of Kinanthropometry and expressed as the mean ± SEM. Anthropometrics parameters and cardiopulmonary exercise test were performed 3–21 days before marathon race and 3–15 after race using a treadmill protocol. The test was performed in 1% fixed slope and speed began with 8 km/h increasing 1 km/h per minute until maximal exhaustion of the runner. Expired gas analysis was performed in a breath-by-breath system (Ergostik®, Geratherm, Bad Kissingen, Germany). Blood samples (30 ml) and urine (20–50 ml) were collected before; immediately after; and 1, 3, and 15 after the marathon race.

### Blood Samples

Blood samples were collected from the antecubital vein in vacuum tubes. Urine, hematological, and iron metabolism analyses were evaluated with routine automated methodology in Clinical Laboratory of Dante Pazzanese Institute of Cardiology immediately after collection. TRIZOL reagent was added in one vacuum tube and stored at −80°C for later genetic analysis. Genetic analysis of *ACTN3* was performed at the Center for Research and Molecular Diagnosis of Genetic Diseases at Federal University of São Paulo.

### Hematological Markers and Iron Metabolism

Plasma iron, creatinine, and bilirubin analyses were performed by colorimetric method; hematological markers hemoglobin (Hb), hematocrit (Ht), red blood cell distribution width (RDW), mean corpuscular volume (MCV), mean corpuscular hemoglobin (MCH), and mean corpuscular hemoglobin concentration (MCHC) were measured by cytochemical/isovolumetric method; and levels of ferritin and myoglobin were evaluated by chemiluminescence assay, erythropoietin by immunochemiluminometric assay, and transferrin by immunoturbidimetry. Hematuria and leukocyturia were analyzed by flow cytometry.

### *ACTN3* Analyses

*ACTN3* genotypes were determined by allelic discrimination assay (TaqMan® SNP Genotyping Assays, Applied Biosystems, Foster City, CA, USA) using Applied Biosystems™ 7,500 Real-Time PCR Systems equipment (Applied Biosystems, Foster City, CA, USA).

### Statistical Analyses

Statistical analyses were performed using ordinary one-way ANOVA and Holm-Sidak’s multiple comparisons *post hoc* test for comparison before and after the race (immediately after, 1 day after, 3 days after, and 15 days after the race) in different genotypes (RR, RX, and XX). Statistical significance was set at *p* < 0.05 (GraphPad Prism version 6.01 software, CA, USA). The values are presented by mean ± standard error mean of 16 XX runners, 43 RX runners, and 22 RR runners and in percentage of runners in RR, RX, and XX genotypes.

## Results

The general and training characteristics of amateur runners are summarized as follows: age, 39.0 ± 1.0 years; height, 1.74 ± 0.1 m; body mass, 74.0 ± 1.0 kg; % of fat mass, 20.0 ± 1.0%; body mass index, 25.0 ± 0.3 kg/m^2^; average training race, 56.0 ± 2.0 km/week; training experience, 6.0 ± 0.5; frequency of training, 4.4 ± 0.7 times/week; time on 10 km race, 46.0 ± 0.7 min. The genotype frequency distribution was 27.2% (*n* = 22) RR, 53.1% (*n* = 43) RX, and 19.8% (*n* = 16) XX genotypes, consistent with the Hardy-Weinberg equilibrium. No significant differences were observed in general and training characteristics among the genotypes (Table 1). During the cardiopulmonary exercise test, speed and oxygen consumption in the anaerobic threshold, respiratory compensation point, and peak were similar before and after marathon race, as well as, among the genotypes (Table 2).

**Table 1:** General and training characteristics of marathon runners separated by genotype.

*ACTN3* R577X	RR	RX	XX
Age (years)	37 ± 2.1	40 ± 1.2	38 ± 1.9
Body mass (kg)	71.6 ± 8.8	77.4 ± 10	70.1 ± 8.5
Height (cm)	1.71 ± 0.0	1.75 ± 0.1	1.73 ± 0.1
Training experience (years)	6.14 ± 4.3	5.6 ± 4.7	7.15 ± 5.1
Training (km/week)	62.2 ± 13	54.2 ± 22	56.1 ± 19.4
Frequency of training (time/week)	4.4 ± 1.2	4.5 ± 1.4	3.9 ± 1
Time of race (min)	254 ± 11	261 ± 6	257 ± 6
10-km race (min)	44.8 ± 5	46.7 ± 6.8	45.7 ± 5.2

*The values presented are the mean ± SEM of 22, 43, and 16 runners in RR, RX, and XX genotypes, respectively*.

**Table 2:** Cardiopulmonary exercise test in the RR, RX, and XX genotypes.

	RR		RX		XX	
	Before	After	Before	After	Before	After
AT speed (km/h)	9.3 ± 0.2	9.3 ± 0.2	9.6 ± 0.3	9.4 ± 0.3	9.2 ± 0.1	9.1 ± 0.1
Respiratory compensation speed (km/h)	17.2 ± 0.5	17.1 ± 0.6	16.5 ± 0.4	16.5 ± 0.4	16.5 ± 0.5	17.1 ± 0.5
Exhaustion speed (km/h)	19.3 ± 0.4	19.3 ± 0.5	18.4 ± 0.4	18.6 ± 0.4	18.2 ± 0.5	18.9 ± 0.6
VO_2_ AT (ml kg^−1^ min^−1^)	32.4 ± 0.8	32.5 ± 0.8	32.4 ± 0.7	30.8 ± 0.6	33.6 ± 1.0	31.4 ± 0.8
VO_2RCP_ (ml kg^−1^ min^−1^)	55.1 ± 1.5	53.2 ± 1.4	51.6 ± 0.9	50.7 ± 0.9	54.3 ± 1.9	54.4 ± 1.6
VO_2peak_ (ml kg^−1^ min^−1^)	58.3 ± 1.5	56.5 ± 1.4	56.6 ± 0.9	54.5 ± 0.9	59.2 ± 1.9	57.0 ± 1.5

*The values are presented as mean ± SDM of 22, 43, and 16 runners in RR, RX, and XX genotypes, respectively. AT, anaerobic threshold; RCP, respiratory compensation point; VO_2_, oxygen consumption*.

Immediately after racing, there was an increase of 40-fold in hematuria (*p* = 4.0e−6), 2% in transferrin (*p* = 1.3e−5), 1% in MCHC (*p* = 0.0005), 40% in creatinine (*p* < 1.0e−15), 23-fold in myoglobin (*p* < 1.0e−15), and 33% in direct bilirubin (*p* = 1.2e−5) and a decrease of Ht (*p* = 0.031) compared to pre-race levels (Table 3). One day after the race, there was a further decrease in erythrocytes (by 6%, *p* = 3.6e−7), Hb (by 6%, *p* = 6.5e−9), and Ht (by 6%, *p* = 3.4e−11), which is accompanied by an elevation of iron (by 20%, *p* = 0.001) and RDW (by 2%, *p* = 0.035). Indirect bilirubin level was stable at 33% above baseline (*p* = 0.0014) and transferrin saturation was increased by 23% (*p* = 1.1e−4) (Table 3). Three days after the race, erythrocytes (*p* = 1.0e−7), Hb (*p* = 6.5e−9), and Ht (*p* = 2.5e−6) remained altered and iron (by 19%, *p* = 0.0023), transferrin saturation (*p* = 0.038), transferrin levels (by 6%, *p* = 0.008) and MCHC (*p* = 0.0005) decreased and RDW (by 2.4% *p* = 0.012) and erythropoietin (by 33%, *p* = 0.0014) increased, suggesting a compensatory response (Table 3). Fifteen days after race, erythrocytes (*p* = 1.610e−4), transferrin saturation (*p* = 0.046), iron levels (*p* = 0.03), and Ht (*p* = 4.9e−6) remained low compared to baseline while MCH and MCHC were still 3–4% higher (*p* = 0.0017 and *p* < 1.0e−15, respectively) (Table 3).

**Table 3:** Hematological changes and iron metabolism after marathon race.

	Before	After	1 day after	3 days after	15 days after
Erythrocytes (10^6^/mm^3^)	5.3 ± 0.04	5.2 ± 0.04	5.0 ± 0.05^b^	4.9 ± 0.05^b^	5.1 ± 0.05^a^
Hb (g/dl)	15.2 ± 0.1	15.1 ± 0.1	14.3 ± 0.1^b^	14.3 ± 0.1^b^	15.0 ± 0.1
Ht (%)	46.9 ± 0.3	45.9 ± 0.3^a^	44.1 ± 0.3^b^	44.6 ± 0.3^b^	44.7 ± 0.3^b^
MCV (fl)	88.1 ± 0.5	88.0 ± 0.5	88.0 ± 0.6	90.0 ± 0.6	88.1 ± 0.6
MCH (pg)	28.6 ± 0.2	28.9 ± 0.2	28.5 ± 0.2	28.8 ± 0.2	29.6 ± 0.2^a^
MCHC(g/dl)	32.4 ± 0.1	32.8 ± 0.1^b^	32.4 ± 0.1	32.0 ± 0.1^b^	33.5 ± 0.1^b^
RDW (%)	12.5 ± 0.1	12.7 ± 0.1	12.7 ± 0.1^a^	12.8 ± 0.1^a^	12.5 ± 0.1
Iron (μg/dl)	112 ± 4.6	119 ± 4.6	134 ± 4.8^a^	91 ± 3.6^a^	97 ± 3.8^a^
Ferritin (ng/ml)	129 ± 10	162 ± 13	149 ± 10	131 ± 9	124 ± 10
Transferrin (mg/dl)	252 ± 3	274 ± 4^b^	247 ± 3	238 ± 3^a^	246 ± 3
Transferrin sat. (%)	44.7 ± 1.8	43.7 ± 1.6	55.1 ± 2.1^b^	38.6 ± 1.6^a^	39.1 ± 1.6^a^
Erythropoietin (mUI/L)	10.0 ± 0.4	10.8 ± 0.5	12.5 ± 1.2	13.3 ± 0.8^a^	11.5 ± 0.7
Hematuria (number/ml)	1,225 ± 89	51,198 ± 15,427^b^	4,052 ± 1,485	2,789 ± 972	2,395 ± 999
TB (mg/dl)	0.9 ± 0.1	1.1 ± 0.1	1.1 ± 0.1	0.8 ± 0.0	0.8 ± 0.0
DB (mg/dl)	0.3 ± 0.0	0.4 ± 0.0^b^	0.3 ± 0.0	0.3 ± 0.0	0.4 ± 0.0
IB (mg/dl)	0.6 ± 0.1	0.7 ± 0.1	0.8 ± 0.0^a^	0.5 ± 0.0	0.5 ± 0.0
Creatinine (mg/dl)	1.0 ± 0.01	1.4 ± 0.04^b^	1.0 ± 0.02	0.9 ± 0.02	0.9 ± 0.02
Myoglobin (ng/ml)	46 ± 6	1,048 ± 78^b^	184 ± 18^a^	69 ± 12	71 ± 17

*Hb, hemoglobin; Ht, hematocrit; MCV, mean corpuscular volume; MCH, mean corpuscular hemoglobin; MCHC, mean corpuscular hemoglobin concentration; RDW, red cell distribution width; TB, total bilirubin; DB, direct bilirubin; IB, indirect bilirubin. The values are presented as mean ± SEM of 22, 43, and 16 runners in RR, RX, and XX genotypes, respectively*.

a *p* < 0.05 vs. before the race;

b **p* < 0.001 vs. before the race*.

**Figure 1:**
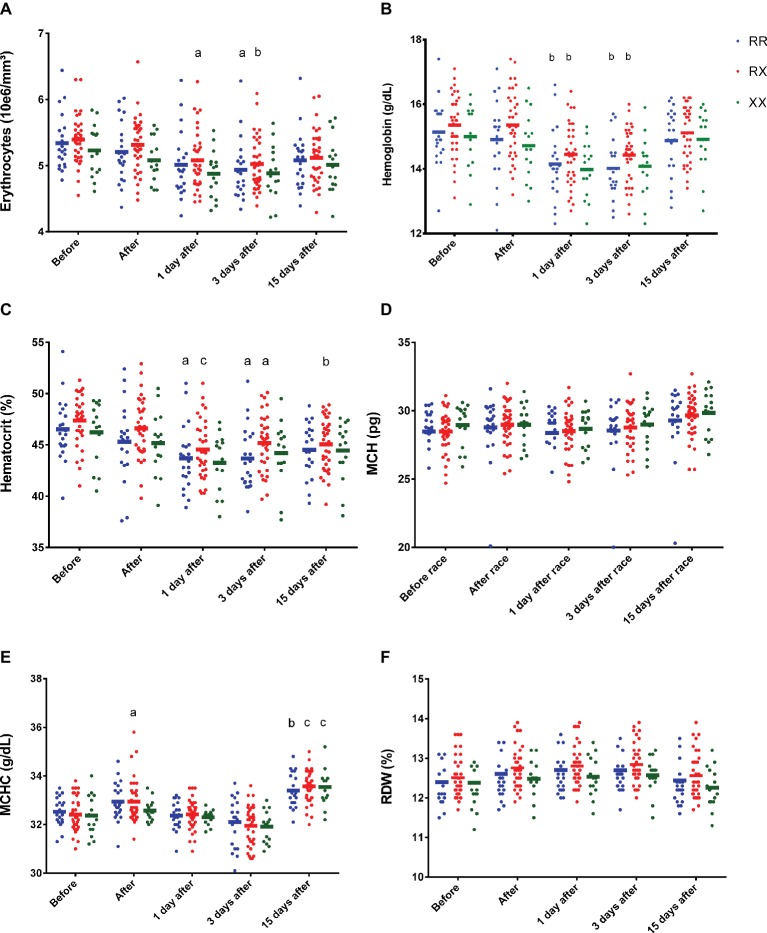
Effect of marathon on hematological markers in RR, RX, and XX genotypes. The hematological markers evaluated were erythrocytes **(A)**; Hb, hemoglobin **(B)**; Ht, hematocrit **(C)**; MCH, mean corpuscular hemoglobin **(D)**; MCHC, mean corpuscular hemoglobin concentration **(E)**; and RDW, red cell distribution width **(F)**. The values are presented as mean ± SEM of 22, 43, and 16 runners in RR, RX, and XX genotypes, respectively. ^a^*p* < 0.05 vs. before race, ^b^*p* < 0.001 vs. before race, ^c^*p* < 0.0001 vs. before race.

There were no genotype differences in the effect of marathon race on erythrocytes, Hb, Ht, MCV, MCH, CHCM, and RDW response among RR, RX, and XX genotypes (Figure 1). However, 1 day after race, there was a decrease in erythrocytes number and an increase in MCHC in RX genotypes (*p* = 0.02 and *p* = 0.018, respectively) (Figures 1A,E,F), and a reduction of Hb and Ht in RR (*p* = 0.006 and *p* = 0.01, respectively) and RX genotypes (*p* = 0.0015 and *p* = 0.0004, respectively) (Figures 1B,C), while XX genotypes remained at baseline levels for hematological parameters (Figure 1). Three days after race, we observed a decrease in erythrocytes, Hb, and Ht in RR (*p* = 0.019, *p* = 0.0018 and *p* = 0.016, respectively) and RX (*p* = 0.0031, *p* = 0.0018 and *p* = 0.034, respectively) genotypes (Figures 1B,C), while they remained at baseline levels in XX genotypes (Figure 1). MCHC elevated 15 days after race in all genotypes (Figure 1E).

**Figure 2:**
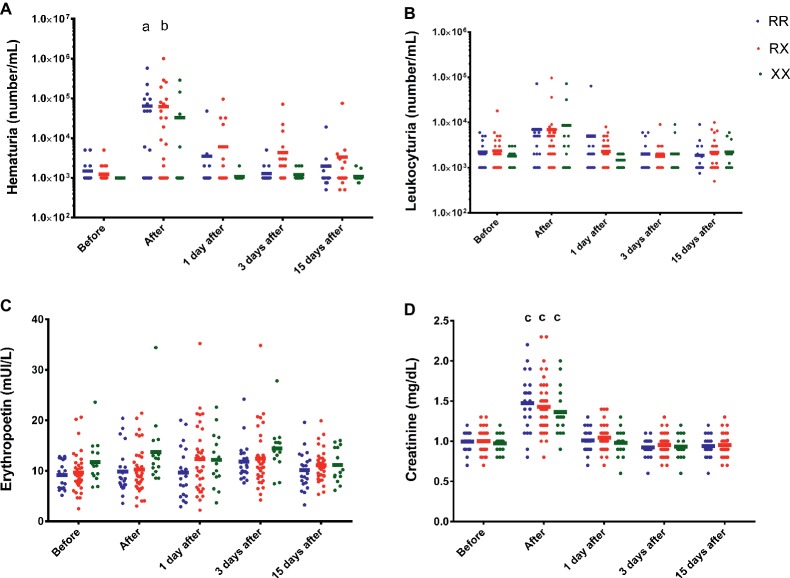
Effect of marathon on hematuria **(A)**, leukocyturia **(B)**, erythropoietin **(C)**, and creatinine levels **(D)** in RR, RX, and XX genotypes. *p* < 0.0001 vs. before race. The values are presented as mean ± SEM of 22, 43, and 16 runners in RR, RX, and XX genotypes, respectively. ^a^*p* < 0.05 vs. before race, ^b^*p* < 0.001 vs. before race, ^c^*p* < 0.0001.

Immediately after race, the results also demonstrated hematuria in RR and RX (*p* = 0.038 and *p* = 0.0039), but not significantly in XX participants (*p* > 0.9999) (Figure 2A). There were no genotype differences in the leukocyturia, creatinine, and erythropoietin levels (Figures 2B–D).

**Figure 3:**
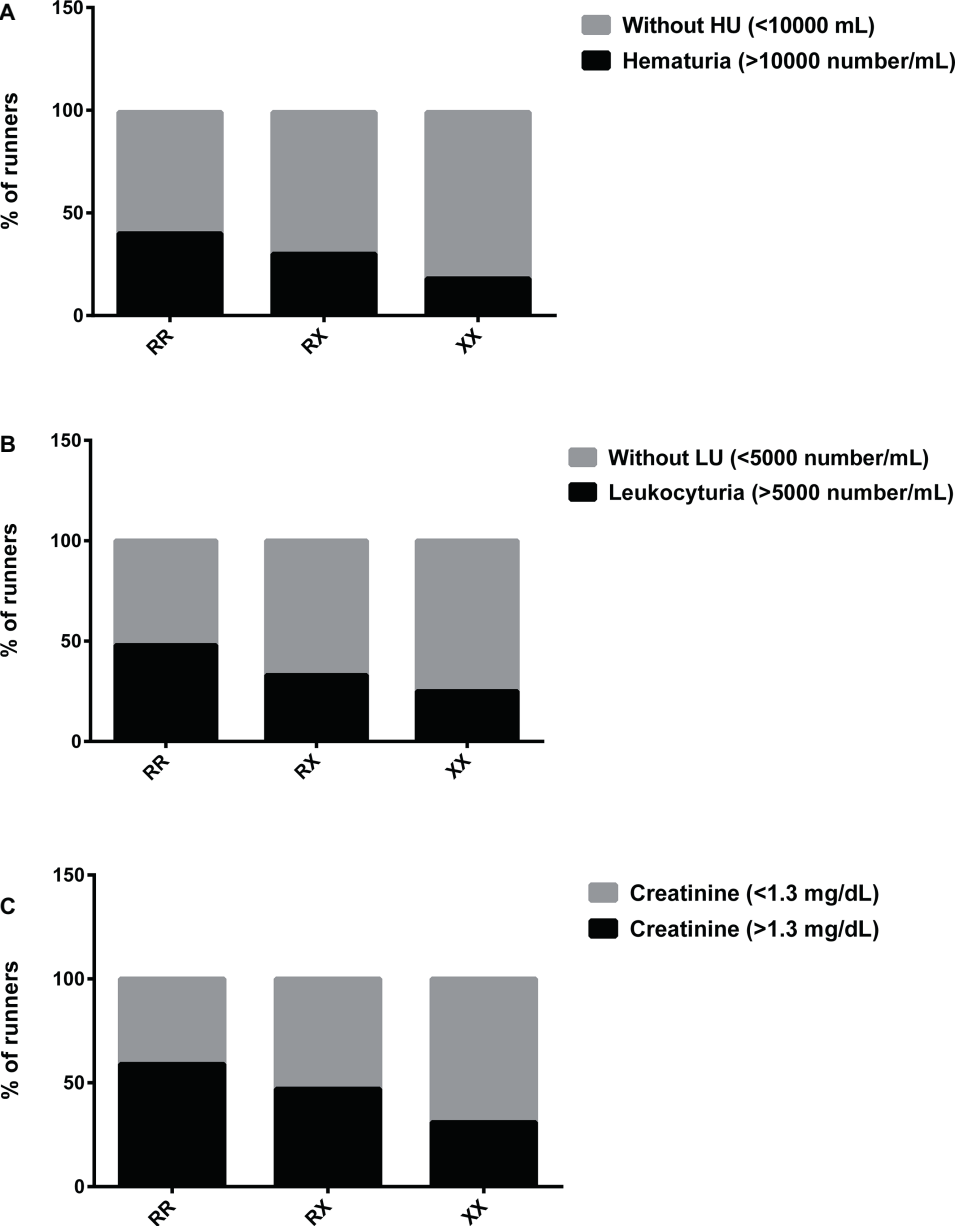
Percentage of runners with hematuria **(A)**, leukocyturia **(B)**, and abnormal creatinine levels **(C)** after marathon race in XX, RX, and RR genotypes. HU, Hematuria; LU, leukocyturia. The values are presented as percentage of 22, 43, and 16 runners in RR, RX, and XX genotypes, respectively.

However, the percentage of runners with hematuria (>10,000 red blood cells/ml) and leukocyturia (>5,000 white blood cells/ml) was higher in RR (40 and 47%, respectively) compared to XX genotype (18 and 25%, respectively) immediately after race (Figures 3A,B). Creatinine (>1.3 mg/dl) imbalance was also higher in RR (59%) compared to XX genotype (31%), suggesting renal dysfunction (Figure 3C).

**Figure 4:**
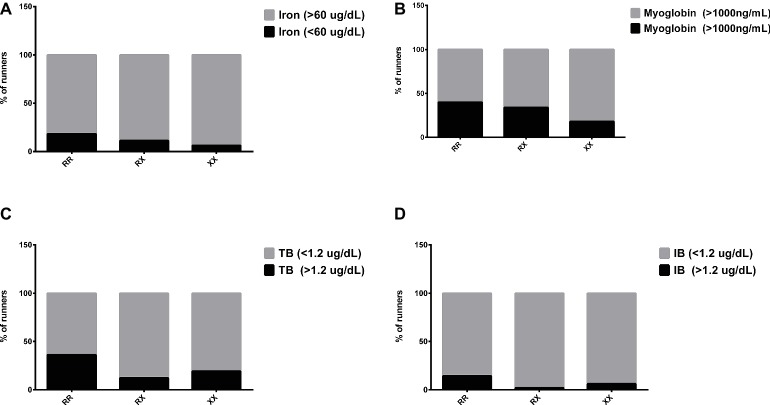
Percentage of runners with abnormal levels of iron **(A)**, myoglobin **(B)**, total bilirubin **(C)**, and indirect bilirubin **(D)** after marathon race in XX, RX, and RR genotypes. The values are presented as percentage of 22, 43, and 16 runners in RR, RX, and XX genotypes, respectively.

Transferrin levels increased 1 day after race in RX genotypes (*p* = 0.004) and the iron levels decreased 3 days after race in RR genotypes (*p* = 0.031), while they remained at baseline levels in XX genotypes (Table 4). In addition, 3 days after race, the percentage of runners with iron deficiency (<60 mg/dl) was higher in RR (18%, 4/22 runners) compared to XX genotype (6.25%, 1/16 runner) (Figure 4A).

**Table 4:** Iron metabolism after marathon race in RR, RX, and XX genotypes.

		Before	After	1 day after	3 days after	15 days after
Iron (μg/dl)	RR	118 ± 5.2	119 ± 5.4	131 ± 4.8	81 ± 2.7^a^	95 ± 4.1
	RX	109 ± 4.2	116 ± 4.3	132 ± 4.3	89 ± 3.3	94 ± 3.4
	XX	110 ± 4.8	129 ± 4.4	144 ± 5.4	110 ± 4.1	106 ± 4
Ferritin (ng/ml)	RR	153 ± 9.3	183 ± 11	172 ± 9.5	151 ± 7.6	142 ± 7.9
	RX	123 ± 9.7	158 ± 14	146 ± 10.8	127 ± 9.5	122 ± 11
	XX	111 ± 8.8	142 ± 14	124 ± 8.4	112 ± 7.6	102 ± 6.9
Transferrin (mg/dl)	RR	245 ± 3	264 ± 4	239 ± 2	231 ± 3	236 ± 3
	RX	252 ± 3	276 ± 4^b^	248 ± 3	238 ± 3	248 ± 2
	XX	261 ± 3	281 ± 5	255 ± 3	246 ± 3	257 ± 3
Transferrin sat. (%)	RR	48 ± 1.9	45 ± 2.1	56 ± 2.2	36 ± 1.3	40 ± 1.6
	RX	44 ± 1.8	42 ± 1.5	54 ± 2	38 ± 1.6	38 ± 1.5
	XX	41 ± 1.6	45 ± 1.1	57 ± 2	45 ± 1.5	41 ± 1.4
TB (mg/dl)	RR	1.1 ± 0.1	1.2 ± 0.1	1.2 ± 0.1	0.8 ± 0.0	0.9 ± 0.0
	RX	0.8 ± 0.0	1.0 ± 0.0	1.0 ± 0.0	0.7 ± 0.0	0.8 ± 0.0
	XX	0.7 ± 0.0	1.0 ± 0.0	1.1 ± 0.0	0.8 ± 0.0	0.9 ± 0.0
DB (mg/dl)	RR	0.5 ± 0.05	0.7 ± 0.06^d^	0.6 ± 0.06^d^	0.5 ± 0.05^c^	0.6 ± 0.1^c^
	RX	0.3 ± 0.01	0.4 ± 0.02	0.3 ± 0.01	0.3 ± 0.02	0.4 ± 0.0
	XX	0.3 ± 0.02	0.4 ± 0.03	0.3 ± 0.01	0.3 ± 0.01	0.4 ± 0.0
IB (mg/dl)	RR	0.8 ± 0.1	0.8 ± 0.1	0.9 ± 0.1^c^	0.5 ± 0.0	0.5 ± 0.0
	RX	0.5 ± 0.0	0.6 ± 0.0	0.7 ± 0.0^c^	0.4 ± 0.0^c^	0.4 ± 0.0
	XX	0.4 ± 0.0	0.6 ± 0.0	0.8 ± 0.0	0.5 ± 0.0	0.5 ± 0.0
Myoglobin (ng/ml)	RR	59 ± 19	1,046±134^d^	147 ± 26	54 ± 8	68 ± 24
	RX	43 ± 4	1,099 ± 112^d^	195 ± 26	58 ± 5	80 ± 32
	XX	38 ± 3	911 ± 184^d^	206 ± 46	116 ± 54	56 ± 7

*TB, total bilirubin; DB, direct bilirubin; IB, indirect bilirubin. The values are presented as mean ± SEM of 22, 43, and 16 runners in RR, RX, and XX genotypes, respectively*.

a *p* < 0.05 vs. before the race;

b *p* < 0.001 vs. before the race;

c *p* < 0.05 vs. XX genotypes;

d **p* < 0.001 vs. XX genotypes*.

The DB concentration was higher in RR genotypes compared to XX genotypes in all periods after the marathon (*p* = 0.0008, *p* < 0.0001, *p* = 0.0167, and *p* = 0.049). There was no genotype difference in the myoglobin levels (Table 4). The percentage of runners with impairment of hemoglobin degradation metabolites, myoglobin (>1,000 ng/ml), TB, and IB (>1.2 μg/ml) was also enhanced in RR (40, 36 and 14%, respectively) compared to XX genotype (19, 19 and 6%, respectively) (Figures 4B–D). Similarly, the percentage of runners with suboptimal levels of ferritin (<100 mg/dl) was higher in RR (62.5%) compared to XX (18.8%) genotype.

## Discussion

Our findings indicate that marathon running induces hemolysis and hematuria and that the body compensates for this by producing erythropoietin and elevating MCHC and MCH in the recovery period. In spite of the compensatory response, hematological parameters (erythrocytes and Ht) and iron levels remained low 15 days after race. Iron levels and hematological parameters seem to be less affected in the runners carrying XX genotype when compared to RR after long-distance exercise. We show in our preliminary results that marathoners with the *ACTN3* XX genotype have reduced susceptibility to hematuria and iron deficiency. Despite these findings being based on a small sample, they raise the possibility of an important role for ACTN3 genotype in marathon performance and, therefore, this investigation should be further replicated.

Decreases in iron concentration between days 3 and 6 are caused by hemolysis, hematuria (25%), gastrointestinal blood loss (10%), and by sweating (4.5 micrograms iron/dl sweat) after long-distance running (Magnusson et al., 1984a,b; Selby and Eichner, 1986; Miller et al., 1988; Seiler et al., 1989; Kratz et al., 2002; Robinson et al., 2006). Iron is transported from the bloodstream bonded to transferrin and the excess intracellular iron is stored in ferritin. It was previously suggested that increased erythrocyte turnover could prevent iron deficiency in endurance athletes (Weight et al., 1991; Peeling et al., 2008). In our study, we demonstrated a decrease in erythrocytes, Hb, and Ht, which is accompanied by an elevation of iron, transferrin saturation, and indirect bilirubin 1 day after race. However, this is followed by a reduction in blood iron, transferrin levels, and saturation and an increase in erythropoietin levels in the recovery period (3 or 15 days after race), consistent with the reported acute iron deficiency in marathon runners during this time period.

Despite the early compensatory increases in erythropoietin, our findings indicate that erythrocytes and Ht remain low compared to baseline 15 days after race, indicating an incomplete hematological system recovery in endurance runners after the conclusion of the race. Peak oxygen consumption is known to decrease after marathon (Sierra et al., 2016) but whether erythrocyte number and Ht contribute to the reduction in oxygen transport and consumption remains unclear. Endurance capacity seems to have high correlation with hemoglobin mass but a low correlation with hematocrit in elite athletes. A systematic review and meta-analysis show that Hb reduction by around 4–7% (after standard blood donation) promotes 7% lower maximal oxygen uptake and 10% lower maximal exercise capacity (Van Remoortel et al., 2017). In our study, Hb returned to baseline levels, while MCH and MCHC increased by 15 days after a marathon that may contribute to improve erythrocyte oxygen release as a compensatory response for low erythrocyte number.

The transferrin receptor activity, electrolytes levels, temperature, and pH interfere with iron release to the cells (He et al., 2000). Iron deficiency with or without anemia may cause a decline in work capacity, reducing delivery of oxygen and aerobic energy system of the working skeletal muscle (Peeling et al., 2008). ACTN3 deficiency seems to be associated with benefits to muscle metabolism in endurance runners (MacArthur and North, 2007). *Actn3* knockout mice have lower muscle mass and strength, IIb fiber diameter, as well as enhanced recovery from fatigue due to “slowing” of the fast muscle fiber profile, with increased aerobic enzymes activity, glycogen accumulation, calcineurin activity, calcium release and absorption by sarcoplasmic reticulum (SR), as well as reduced glycogen phosphorylase and lactate dehydrogenase activity (MacArthur et al., 2007; Chan et al., 2011; Lee et al., 2016). Tissue oxygen supply during exercise depends on temperature, pH, and some products of oxidative metabolism such as CO_2_; ATP; 2,3-DPG; and Cl^−^ (Mairbaurl et al., 2013). The association of *ACTN3* polymorphism and parameters of tissue oxygen supply, such as hematological markers and iron metabolism after exercise has not been investigated. We did not observe differences in hematological response between RR, RX, and XX genotypes, but significantly higher hematuria and iron reduction were observed only in RR and/or RX genotypes but not in the XX group. Further studies are needed to determine the association between hematuria and *ACTN3* polymorphism due to low sample size of this study. Moreover, we observed a higher percentage of runners with leukocyturia and hematuria in RR compared to XX genotype. Exhaustive exercise may also enhance serum creatinine to concentrations >0.3 mg/dl or >50% over baseline, indicating acute renal dysfunction (Shephard, 2016).

The potential mechanisms of renal injury, demonstrated by hematuria, leukocyturia, and creatinine levels are tract trauma, hypoxic renal injury and ischemia, release of a hemolysing factor during exercise, dehydration, products of hemolysis, myoglobinuria release, and peroxidation of red blood cells (Bellinghieri et al., 2008; Akiboye and Sharma, 2018). In our study, we also observed a higher percentage of runners with impairment of hemolysis metabolites and myoglobinuria with the RR genotype compared to those with the XX genotype. Hematuria also could be attributed to bladder trauma, foot strike, presence of dysmorphic red blood cells, acidosis, and hypoxia-related glomerular injury (Bellinghieri et al., 2008; Shephard, 2016; Grygorczyk and Orlov, 2017; Akiboye and Sharma, 2018).

We also observed lower percentage of runners with iron deficiency in XX genotype. The main marker of erythrocyte turnover is the hormone erythropoietin, which was higher in XX genotypes, suggesting that the small number of XX runners with iron deficiency were also influenced by increased erythrocyte turnover after race. However, the efficient turnover could contribute to higher prevalence of runners with suboptimal ferritin levels (<100 μg/L) in the XX group, but not ferritin and iron deficiency. A known contributing factor to iron and ferritin status is the level of hepcidin, which is a key regulator of iron homeostasis. Peeling et al. (2014) reported that athletes with ferritin levels <30 μg/L are unlikely to present with increased hepcidin post-exercise. In our study, all the athletes presented with ferritin values above 30 μg/L.

## Conclusion

Hematological changes induced by long-distance running exercise could impair performance and health, increasing the risk of sports anemia and renal failure. In conclusion, our results show that *ACTN3* polymorphism may in part explain different hematological responses in endurance athletes. This is the first study that suggested that RR individuals seem to be more susceptible to hemolytic anemia and hematuria and that supplementation and renal function evaluation should be monitored. These results are based on a small sample and should be further replicated. Strategies to improve erythrocytes turnover in endurance athletes could be proposed.

## Ethics Statement

Subjects were informed of the experimental procedures and possible risks and signed a term of informed consent before participating, approved by the Ethics Committee of Dante Pazzanese Institute of Cardiology, Brazil (Permit Number: 979/2010), in accordance with the Declaration of Helsinki.

## Author Contributions

AS designed and conducted research, analyzed the data, and performed statistical analysis. RO, ES, GL, and MB analyzed the data, conducted research, and revised the manuscript. NG, CS, MK, and JP designed and conducted research and revised the manuscript. JS revised the manuscript. MC-B designed and conducted research, performed statistical analysis, revised the manuscript, and had primary responsibility for the final content. All authors have read and approved the final version of the manuscript, and agree with the order of presentation of their names.

### Conflict of Interest Statement

The authors declare that the research was conducted in the absence of any commercial or financial relationships that could be construed as a potential conflict of interest.
